# Health data collection methods and procedures across EU member states: findings from the InfAct Joint Action on health information

**DOI:** 10.1186/s13690-021-00780-4

**Published:** 2022-01-05

**Authors:** Brigid Unim, Eugenio Mattei, Flavia Carle, Hanna Tolonen, Enrique Bernal-Delgado, Peter Achterberg, Metka Zaletel, Stefanie Seeling, Romana Haneef, Anne-Charlotte Lorcy, Herman Van Oyen, Luigi Palmieri

**Affiliations:** 1 Department of Cardiovascular, Endocrine-metabolic Diseases and Aging, Istituto Superiore di Sanità, Via Giano della Bella 34, 00162 Rome, Italy; 2 Center of Epidemiology, Biostatistics and Medical Information, Marche Polytechnic University, Ancona, Italy; 3 Department of Public Health and Welfare, Finnish Institute for Health and Welfare (THL), Helsinki, Finland; 4 Data Sciences for Health Services and Policy Research Group, Institute for Health Sciences in Aragon (IACS), Zaragoza, Spain; 5 Centre for Health Knowledge Integration, National Institute for Public Health and the Environment (RIVM), Bilthoven, Netherlands; 6 Health Data Centre, National Institute of Public Health, Ljubljana, Slovenia; 7 Department of Epidemiology and Health Monitoring, Robert Koch Institute, Berlin, Germany; 8 Department of Non-Communicable Diseases and Injuries, Santé Publique France, 94415 Saint-Maurice, France; 9 Directorate of Health, Luxembourg, Luxembourg; 10 Epidemiology and Public Health, Sciensano, Brussels, Belgium

**Keywords:** Health data, Health information, Data collection methods, Quality assessment, Data availability, Data accessibility, FAIR principles

## Abstract

**Background:**

Health-related data are collected from a variety of sources for different purposes, including secondary use for population health monitoring (HM) and health system performance assessment (HSPA). Most of these data sources are not included in databases of international organizations (e.g., WHO, OECD, Eurostat), limiting their use for research activities and policy making. This study aims at identifying and describing collection methods, quality assessment procedures, availability and accessibility of health data across EU Member States (MS) for HM and HSPA.

**Methods:**

A structured questionnaire was developed and administered through an online platform to partners of the InfAct consortium form EU MS to investigate data collections applied in HM and HSPA projects, as well as their methods and procedures. A descriptive analysis of the questionnaire results was performed.

**Results:**

Information on 91 projects from 18 EU MS was collected. In these projects, data were mainly collected through administrative sources, population health interview or health examination surveys and from electronic medical records. Tools and methods used for data collection were mostly mandatory reports, self-administered questionnaires, or record linkage of various data sources. One-third of the projects shared data with EU research networks and less than one-third performed quality assessment of their data collection procedures using international standardized criteria. Macrodata were accessible via open access and reusable in 22 projects. Microdata were accessible upon specific request and reusable in 15 projects based on data usage licenses. Metadata was available for the majority of the projects, but followed reporting standards only in 29 projects. Overall, compliance to FAIR Data principles (Findable, Accessible, Interoperable, and Reusable) was not optimal across the EU projects.

**Conclusions:**

Data collection and exchange procedures differ across EU MS and research data are not always available, accessible, comparable or reusable for further research and evidence-based policy making. There is a need for an EU-level health information infrastructure and governance to promote and facilitate sharing and dissemination of standardized and comparable health data, following FAIR Data principles, across the EU.

**Supplementary Information:**

The online version contains supplementary material available at 10.1186/s13690-021-00780-4.

## Introduction

Health data are collected worldwide from various sources for different purposes, including the secondary use for health monitoring (HM), public health surveillance, health system performance assessment (HSPA) and health research in general. HM is an intermittent or episodic performance and analysis of measurements aimed at detecting changes in the health status of populations or in the physical or social context [1]. Population HM, the regular and institutionalized production and dissemination of information and knowledge about the health status of a population, is an essential element of public health [2]. Public health surveillance can be defined as the ongoing systematic collection, analysis, and interpretation of health data, essential to the planning, implementation, and evaluation of public health practice, closely integrated to the dissemination of these data to those who need to know and linked to prevention and control [3]. An important determinant of population health is the performance of national health systems. HSPA aims at monitoring, evaluating and communicating the extent to which various aspects of the health system meet key objectives such as health conferred on citizens by the health system, responsiveness to individual needs and preferences of patients, financial protection offered by the health system and productivity of utilization of health resources [4, 5]. A healthcare system should also fulfil other criteria such as equity on access, effectiveness, quality and safety, and allocative efficiency [6]. Data collected for HM, public health surveillance and HSPA are not always available in databases of international organizations, such as those of the World Health Organization (WHO), Organization for Economic Co-operation and Development (OECD) or the European Statistical Office (Eurostat), limiting their use for research, policy making, international benchmarking and comparisons, and the opportunities for countries to learn from each other [7].

Comparability of research findings is fundamental for comparisons across different geographical areas and over time but is often limited by differences in indicator definitions, data collection methods and tools, and the use of different classifications. Comparability of research results can be ensured through standardization of data collection methods and quality assessment procedures. Standardization of metadata is also important in health information systems for the description of health data, considering that metadata facilitates data comparisons, access to and reuse of public information. Metadata can be defined as “explanatory texts documenting statistical data and providing summary information on definitions of populations, objects, variables, the methodology and quality, and the statistical production process in general” [8].

The present study is part of the Joint Action (JA) on Health Information InfAct (Information for Action), that was launched in 2018 and will end in 2021. Through the collaboration of 40 partners from 28 EU MS and 4 associated countries, the JA works towards a sustainable infrastructure for EU health information that will support evidence-based policy and innovative, high quality research. The aims of this study were to identify and compare data collection methods and related harmonization and quality assessment procedures, and to perform a pilot mapping exercise on availability and accessibility of health data for HM and HSPA in EU MS.

## Methods

Databases fostered by selected international organizations (i.e., Eurostat, WHO-Health For All database, WHO-Health 2020 monitoring framework, WHO-Global non-communicable diseases monitoring framework, and OECD) and EU research networks (i.e., European Community Health Indicator Monitoring System-ECHIM, Joint Assessment Framework on Health- JAF) providing EU health indicators were scoped to analyse their underlying methodologies and procedures. In light of those experiences, a questionnaire (Additional file 1) was designed and administered to representatives and national experts from InfAct partner countries (28 EU MS and 4 associated countries). Further participants were identified through a snowball recruitment process. To this purpose, InfAct partners were asked to forward the questionnaire to national colleagues with good knowledge and experience in HM and HSPA in their country, such as epidemiologists, researchers that have played leading roles in EU projects, health data managers engaged in national health and research institutions, and universities. The questionnaire included the following information:

i) Source of information, types of data sources used (e.g., European Health Interview or Examination Surveys (EHIS-EHES), census, administrative data);
ii) Methodology, tools and approaches for data collection (e.g., questionnaires, face-to-face interviews, medical examination);
iii) Quality assurance procedures and quality dimensions or criteria considered (Additional file 2). The projects were assessed with the quality dimensions or criteria defined by Eurostat (i.e., relevance, accuracy, timeliness, punctuality, comparability, coherence, accessibility and clarity) [9] in addition to two quality criteria considered by the European Collaboration for Healthcare Optimization-ECHO (coverage and internal reliability) [10];
iv) Availability of microdata (individual records) or macrodata (aggregated results), metadata, and data formats (e.g., digital, printed formats);
v) Accessibility and standard for exchange and sharing of data and metadata (e.g., request and approval required for data access; data are transferable to approved users and reusable; request for financial charge for data access).

The sections of the questionnaire on health data availability and accessibility were developed according to the FAIR Data Principles, which are a set of guiding principles in order to make data *Findable* (data and supplementary materials have sufficiently rich metadata and a unique and persistent identifier); *Accessible* (metadata and data are understandable to humans and machines, and data is deposited in a trusted repository); *Interoperable* (metadata use a formal, accessible, shared, and broadly applicable language for knowledge representation); and *Reusable* (data and collections have a clear usage license and provide accurate information on provenance) [11].

The inclusion criteria for the projects were as follows: i) health data provided by the project should be representative of the population at national or regional level; ii) health data should cover topical areas of population HM and/or HSPA; iii) the project should not focus on rare diseases, infectious diseases or cancer; iv) health data should be accessible as microdata or macrodata but not included in databases of international organizations; and v) the project should have produced scientific outputs (e.g. scientific articles, public reports). Eligible projects could be part of European health research networks (e.g., EHES, ECHIM, ECHO, European Cardiovascular Indicators Surveillance Set-EUROCISS), but the related data or indicators should not be included in databases of international organizations (e.g., WHO-Europe, OECD, Eurostat).

The final version of the questionnaire was administered from June to October 2019 to InfAct partners through the LimeSurvey online platform [12]. A set of definitions was provided to the participants, through an online page, to facilitate comprehension of the survey items (Additional file 2). A descriptive analysis of the questionnaire results was performed using the statistical package SPSS v.26 (IBM SPSS Statistics for Windows, Armonk, NY: IBM Corp).

## Results

### General characteristics

Information about 91 projects (Additional file 3) were collected from 18 EU MS (i.e., Belgium, Croatia, Czech Republic, Estonia, Finland, France, Germany, Italy, Latvia, Luxembourg, the Netherlands, Portugal, Romania, Serbia, Slovenia, Spain, Sweden, and the United Kingdom). The authorities or organizations responsible for the projects were mostly National Institutes of Public Health (25/91), National Health Institutes (17/91), and Universities (14/91). Some identified projects were also research networks, for instance the Burden of Disease Network (BOD), European Perinatal Health Surveillance System (Euro-Peristat), and EHES.

### Characteristics of the sources of information

The 91 projects are representative at national (45/91 projects), regional (20/91), or both levels (26/91). The main objectives of the projects are elaboration of HM indicators (70/91), health data collection (57/91), elaboration of HSPA indicators (30/91), standardization and harmonization of methods and procedures (29/91), and development and/or validation of specific tools (23/91).

The health data sources reported in the projects (Table 1) were mostly administrative data sources (e.g., hospital discharge records, mortality, pharmaceutical prescription) (52/91), followed by EHIS (22/91), electronic medical records (20/91), clinical data registries (19/91) and population-based disease registries (18/91). The period for data collection varied greatly, as shown in Table 1. It was mostly continuous for administrative data (26/52), clinical data registries (17/18), electronic medical records (14/20), population-based disease registries (13/17), hospital-based registries (10/15), and clinical quality registries (4/7). The data collection period was mainly periodic for EHIS (15/22), followed by primary data obtained through direct examination (8/15) or interviews (8/14), longitudinal studies (7/11) and EHES (7/13). A single implementation of data collection was reported for all data sources, except for population-based disease registries and intermediate linked data sources.

**Table 1:** Health data sources used in the projects identified through survey responses

DATA SOURCES	DATA COLLECTION PERIOD				
	N projects	Single collection	Periodic	Continuos	Periodic intervals (years)
Population health examination survey (HES)	13	x	◊	x	3–5
Population health interview survey (HIS)	22	x	◊	x	1–7
Population-based disease registries	17	–	x	◊	1–4
Hospital based registries	15	x	x	◊	Mo; 1–5
Clinical quality registries	7	x	x	◊	5
Medical records/clinical data registries	18	x	x	◊	5
e-health solutions (mhealth devices)	2	x	–	x	–
Longitudinal or cohort study	11	x	◊	x	2–4
Administrative data	52	x	x	◊	Mo; 3–5
Electronic medical/health records	20	x	x	◊	1–5
Intermediate linked data sources	5	–	◊	x	1
Primary data collected by direct examination	15	x	◊	x	2–10
Primary data collected through interview	14	x	◊	–	2–5
Other: Geographic information/geospatial data (3 projects); media data; official statistics (e.g. consumption statistics, school entrance examinations, microcensus, birth statistics, land use statistics); routine data (e.g. quality reports of the health insurance)	6	x	x	◊	4

x, implemented; ◊, most frequent data collection period; Mo, monthly; −-, no data

### Health data collection methods and related procedures

**Fig. 1:**
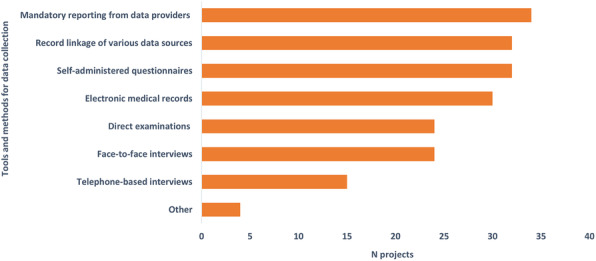
Tools and methods for health data collection

The projects are mostly related to HM (84/91), followed by health system performance monitoring (27/91) and HSPA (21/91). Common tools and methods for health data collection (Fig. 1) were mandatory reporting from data providers (34/91), self-administered questionnaires (32/91), record linkage of various data sources (32/91), and electronic medical records (30/91); 24-h dietary recall (diary type) was also used in few projects.

**Fig. 2:**
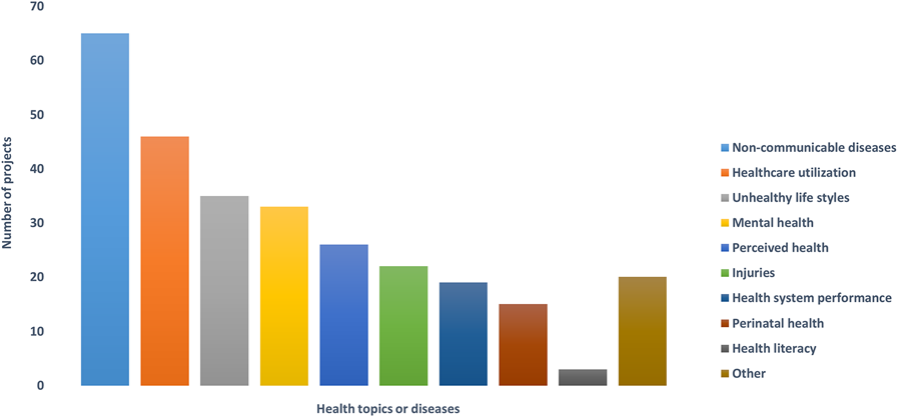
Health topics or diseases considered in the projects

**Fig. 3:**
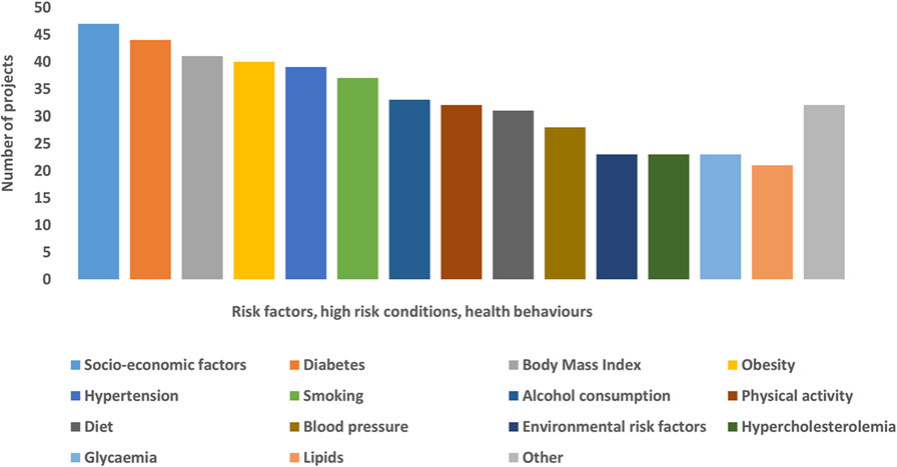
Risk factors, high-risk conditions and health behaviours investigated in the projects

The main health topics or diseases considered in the projects were non-communicable diseases (65/91), healthcare utilization (46/91), unhealthy lifestyles (35/91) and mental health (33/91) (Fig. 2). Information on risk factors, high-risk conditions and/or health behaviours (Fig. 3), such as diabetes (44/91), obesity (40/91) and hypertension (39/91) were also provided. Other risk factors considered were for instance consumption of illicit substances, causes and circumstances of injuries, and cardiovascular events.

The areas defined in the protocols of the projects (Table 2) are mostly related to statistical analysis (78/91), reporting (59/91) and quality data control (55/91). The protocols included internationally recognized standardized methods and procedures in all areas, but mostly for statistical analysis (50/78), quality data control (32/55), reporting (32/59), and laboratory analysis (17/17). The projects adhered in various extent to standardized methods and procedures provided by international organizations (e.g., WHO-Europe, Eurostat) and research networks (e.g., EHES, EHIS, Euro-Peristat) regarding most areas of data management and reporting. For reporting standards, the projects also followed the recommendations of the International Committee of Medical Journal Editors (ICMJE) for the conduct, reporting, editing, and publication of research studies in medical journals.

**Table 2:** Areas defined in the protocols of the identified projects

			Guidelines and recommendations on standardized methods	
Areas defined in the protocol	N projects	Standardized methods are reported in the protocol (N projects)	International organizations/committees	Research networks/EU projects
**Quality data control**	55	32	WHO-Europe, Eurostat, IARC	EHES, EHIS, Euro-Peristat, SHARE-ERIC, HBSC, GBD
**Accessibility**	25	13	WHO-Europe, CDC, IHE Europe	EHES, HBSC, SHARE-ERIC, Atlas VPM
**Availability**	26	13	WHO-Europe, CDC, IHE Europe	EHES, HBSC, SHARE-ERIC, Atlas VPM
**Statistical analysis**	78	50	WHO-Europe, Eurostat, IARC	–
**Laboratory analysis**	17	17	CLSI	EARS-Net
**Reporting**	59	32	WHO, ECDC, ICMJE	EHES, HBSC, INSPIRE
**Data linkage**	38	15	WHO-Europe, Eurostat, IARC	–
**Data sharing**	16	9	–	–

IARC, International Agency for Research on Cancer; Eurostat, European Statistical Office; SHARE-ERIC, Survey on Health, Ageing and Retirement in Europe; HBSC, Health Behaviour in School-aged Children; GBD, Global Burden of Disease; CDC, Centers for Disease Control and Prevention; IHE Europe, Integrating the Healthcare Enterprise; EHES, European Health Examination Survey; Atlas VPM, Spanish National Framework for Information Security for a geospatial project; CLSI, Clinical and Laboratory Standards Institute; EARS-Net, European Antimicrobial Resistance Surveillance Network; ECDC, European Centre for Disease Prevention and Control; INSPIRE, Infrastructure for Spatial Information in Europe; ICMJE, International Committee of Medical Journal Editors; −-, no data

The indicators elaborated from the collected health data were mainly prevalence (59/91), outcome measures (52/91), incidence (47/91), performance measures (25/91), and attack rates (8/91). Other indicators reported by the respondents were sick-leave indicators, synthetic prognostic scores, social and geographical inequalities indicators, temporal trends measures, and burden of disease indicators (disability-adjusted life years; years lived with disability). The elaborated indicators were used for monitoring (73/91), policy planning (66/91), research purposes (66/91) and health services evaluation (30/91). The funding source for the majority of the projects (84/91) was public (e.g., Ministry of Health, Ministry or Research, Italian Medicines Agency, European Food Safety Authority). Other funding sources reported were pharmaceutical companies (e.g., Roche Pharma) and scientific societies (e.g., Italian Society of Neurology).

Health data collected or used by 30 projects were shared with EU research networks and projects (e.g., ECHIM, ECHO, EHES, EHIS, European Best Information Through Regional Outcomes In Diabetes-EUBIROD, Euro-Peristat) (Table 3), while for 4 projects the data sharing process is under development. However, the majority of projects (57/91) do not share data with EU research networks or projects.

**Table 3:** Data collections shared with European projects and research networks

COUNTRY	RESEARCH PROJECT	EU RESEARCH NETWORKS AND PROJECTS
BELGIUM	Health Interview Survey	EHIS
BELGIUM	Belgian Health Examination Study	EHES
BELGIUM	Belgian Treatment Demand Indicator Register	EMCDDA
BELGIUM	Health Status Report	EHES
BELGIUM	Initiative for Quality improvement and Epidemiology in Diabetes	EUBIROD
BELGIUM	Initiative for Quality Improvement and Epidemiology in Children and Adolescents with Diabetes	EUBIROD
CROATIA	CroDiab	ns
FINLAND	Different administrative registries	EuroHOPE
FRANCE	Euro-Peristat	Euro-Peristat coordinating center
GERMANY	German Health Update (GEDA)	EHIS
GERMANY	BURDEN 2020-EHIS	EHIS
ITALY	Health Behaviour in School-aged Children	HBSC network
ITALY	Italian nationwide longitudinal population-based study on DKA at diagnosis of type 1 diabetes	Joint International Project DKA at onset of pediatric type-1 diabetes
ITALY	LINFA project: Longitudinal Infant and Neonatal Follow-up towards Adolescence	EUROCAT, EURORDIS
ITALY	European Injury Database	ECHIM
ITALY	Italian Obstetric Surveillance System (ItOSS)	International network of obstetric survey system (INOSS), Euro-Peristat
ITALY	Drug-related mortality and hospitalization in Italy	EMCDDA, ECHIM
ITALY	Moli-sani Study	MORGAM project, BIOMARCARE Consortium, CHANCES project
LUXEMBOURG	European Injury Data Base	Injury Database Network
LUXEMBOURG	Observation of Cardiovascular risk factors in Luxembourg (ORISCAV-LUX 1 & 2)	NESCAV
LUXEMBOURG	Luxembourg’s Birth-Related Health-Monitoring System (SUSANA)	Euro-Peristat
LUXEMBOURG	European Health Examination Survey	EHES coordinating centre
LUXEMBOURG	Luxembourgish Information System on Drugs and Drug Addiction	EMCDDA, REITOX
LUXEMBOURG	Health Behaviour in School-aged Children	HBSC network
LUXEMBOURG	Neonatal Hearing Screening	EUScreen
LUXEMBOURG	SHARE	SHARE-ERIC
PORTUGAL	National Health Interview Survey	EHES; HBM4EU
ROMANIA	Romanian study	ns
SPAIN	Atlas of Variations in Medical Practice in the Spanish National Health Service (Atlas VPM project)	ECHO
UNITED KINGDOM	Secure Anonymised Information Linkage (SAIL) system	ECHIM, Injury Database Network

EHIS, European Health Interview Survey; EHES, European Health Examination Survey; EMCDDA, European Monitoring Centre for Drugs and Drug Addiction; EUBIROD, European Best Information Through Regional Outcomes In Diabetes; EuroHOPE, European Health Care Outcomes, Performance and Efficiency; Euro-Peristat, European Perinatal Health Surveillance System; HBSC, Health Behaviour in School-aged Children; EUROCAT, European network of population-based registries for the epidemiological surveillance of congenital anomalies; EURORDIS, Rare Diseases Europe; ECHIM, European Community Health Indicator Monitoring System; MORGAM project, MOnica Risk, Genetics, Archiving and Monograph; BIOMARCARE consortium, Biomarker for Cardiovascular Risk Assessment; CHANCES, Consortium on Health and Aging-Network of Cohorts in Europe and the United States; NESCAV, Nutrition, Environment and Cardiovascular Health; REITOX, European information network on drugs and drug addiction; EUScreen, Vision and hearing screening programmes for children in Europe; SHARE-ERIC, Survey of Health, Ageing and Retirement in Europe; HBM4EU, EU Network of Human Biomonitoring Laboratories; ECHO, European Collaboration for Healthcare Optimization; ns, not specified

Regarding quality assurance procedures in health data collection (Additional file 2), the most identified quality dimensions or criteria in the projects were relevance and comparability (65/88 each), followed by coverage (58/88), accuracy (52/88) and internal reliability (47/88). The least reported quality dimensions were punctuality and accessibility (28/88 each). Quality assurance procedures were not reported in three projects.

### Availability of health information

Due to nonresponse (missing data), details on availability of health information are reported for less than 91 projects identified in the study. Collected health data are stored as microdata (41/86), macrodata (12/86), or both (33/86). Most projects with microdata (59/74) have a global unique and eternally persistent identifier or study identifier. Out of 45 projects with macrodata, only 14 have an interactive system for users to perform further data aggregation and/or stratification. The available formats of the collected health data are first of all electronic files (75/86), followed by scientific publications (40/86), websites (33/86) and CD-ROM in one project.

The majority of the projects (50/84) had a publicly available description of the dataset purpose and content or metadata. The metadata followed reporting standards in 29 projects, of which 7 were international reporting standards, such as those defined by Eurostat (Additional file 2), 8 were national reporting standards, and 14 were ad-hoc metadata reporting standards developed for the purpose of a single project. The survey respondents specified few international reporting standards, in particular the Data Documentation Initiative and the Euro SDMX Metadata Structure (Additional file 2); national and ad-hoc metadata reporting standards were not specified.

### Accessibility of health information

**Fig. 4:**
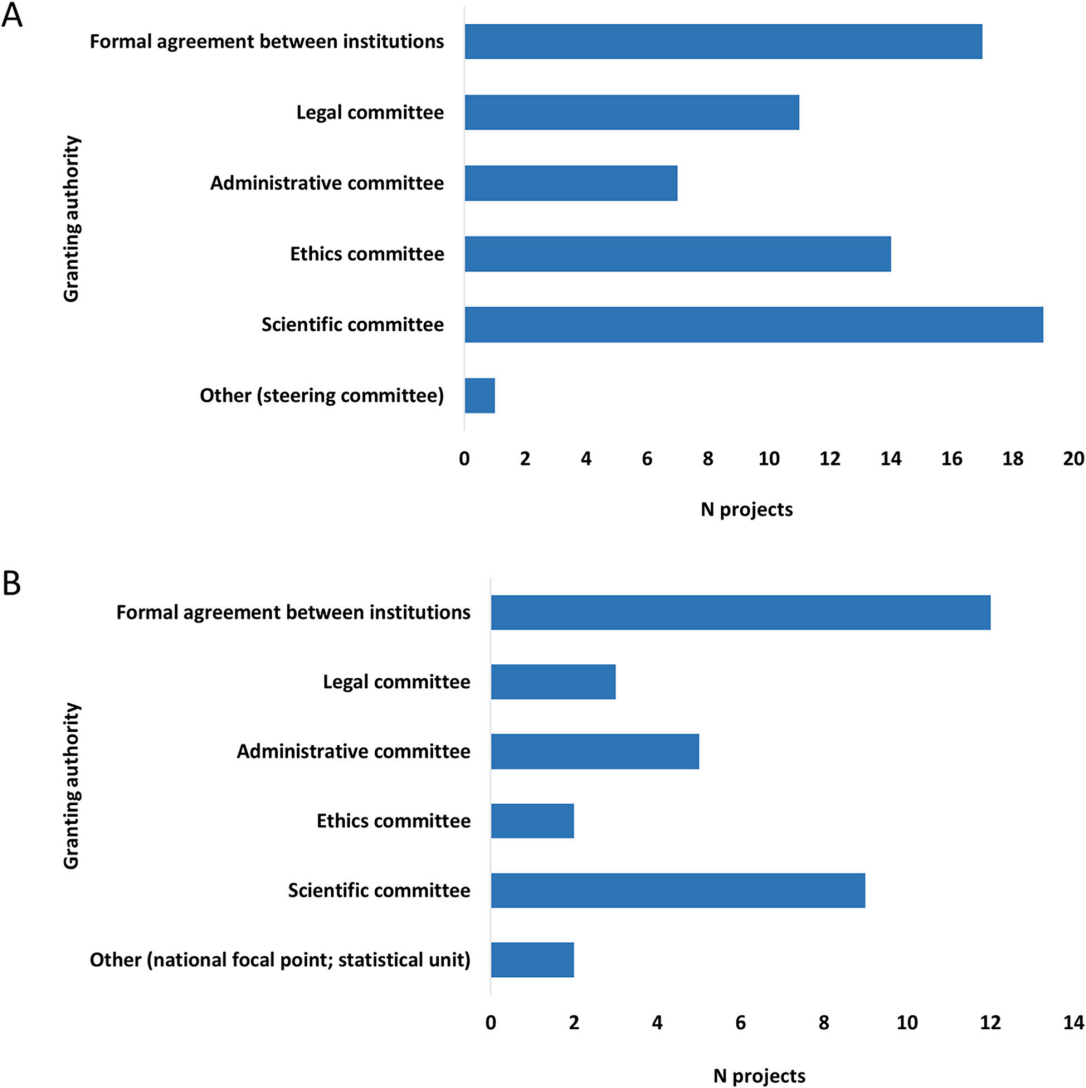
Authorities granting access to microdata (A) and macrodata (B)

Due to nonresponse (missing data), details on accessibility of health information are reported for less than 91 projects identified in the study. Health data were accessible to external users in 34/86 projects, as microdata (21/34) or macrodata (28/34). Microdata were only available to users upon specific request followed by approval, while macrodata were available to all users in open access (22/22) or upon request followed by approval (18/22). The access to microdata or macrodata was mostly granted by scientific committees or through a formal agreement between institutions (Fig. 4). Considering data reusability, microdata were reusable based on a data usage license (e.g., for a specific project, analysis, period of use, private or public use) in 15/21 projects and without a specific license in 4/21 projects. Macrodata were reusable based on a data usage license in all projects (22/22) and for all users in 15/22 projects. A financial charge for data access is not required in most projects (44/60).

## Discussion

This study highlights the heterogeneity in data collection methods and quality assessment procedures and the paucity of available, accessible, internationally comparable or reusable health data and information for research purposes and policy making in and across EU countries. The extensive use of administrative data sources for HM and HSPA observed in the study confirms the increasingly widespread utilization of these data across MS and underlines the importance of access to high quality and nationally representative data. However, adherence to standardized data collection methods and procedures provided by international organizations and EU research networks is not uniform across the identified projects. Moreover, only one-third of the projects share data with EU research networks, quality assurance in data collection is seldom assessed through available quality criteria, and less than half of the projects follow metadata reporting standards for data description. Given the importance of health information for research and policy development, improving health information is therefore a priority in Europe.

Although population health monitoring and surveillance is an essential pillar of public health and most identified projects are related to HM, the development and implementation of public health surveillance systems producing data and indicators comparable between different areas of a country and between EU countries has been slow and inconsistent. For instance, the four behavioural and lifestyle surveillance systems operating in Italy (OKkio alla SALUTE, Health Behaviour in School-aged Children-HBSC, Progress by local health units towards a healthier Italy-PASSI, and Surveillance system in the population over 64 years-PASSI d’Argento) [13–16] have been implemented at different times and the utilization rate of the data and indicators varies across the country, resulting in higher usage in Northern Regions and, partly, in Central Regions. This limits the correct definition of priorities and objectives of public health programs at regional and national level, as well as comparability across Italian Regions and with other EU MS [17]. Sharing of health data and information collected through standardized methods and procedures is an effective way to strengthen public health surveillance systems [7] and to assure that policy planning is based on reliable and accurate data.

The availability and accessibility of health data and information provide several opportunities that are not limited to a possible wider utilization of the datasets but also include the possibility of data linkage across datasets and the development of new indicators used for HM and HSPA. Restrictions in data processing observed in most projects restrict these advantages, including national and international collaborations and partnerships that could enhance research activities and their relevance and outputs. Other important aspects of health datasets are availability and accessibility of metadata and metadata reporting standards; the latter is defined as “the rules by which information about data is recorded in order to facilitate understanding of the origin, derivation, and/or provenance of the data” [18]. Metadata are available and accessible in most identified EU research projects collecting health data. However, the metadata follow international standards only in few projects and, in most cases, are defined according to specific and ad-hoc needs of the projects, limiting the sharing of health data and their secondary use outside the specific project. Data access could also be limited by availability of financial resources which could limit the quality and efficiency of scientific research, especially from developing countries, lower ranked institutions and researchers with limited resources. According to our findings, only 16 projects out of 60 apply a financial charge for data access, but this information is missing for 34% of the total projects.

Improving data access will enhance research activities, reduce inequality and increase the diversity of scientific outputs [19]. These issues are tackled by the EU’s open science policy that promotes open data and open access publications. In addition, the European Open Science Cloud, currently under development, will enable researchers to store, process and share data [20].

Despite the undisputable value of data accessibility and data sharing, there are concerns regarding ethical and legal issues that cannot be disregarded, namely those regarding intellectual property, privacy and confidentiality. Data access in most projects requires approval granted by a scientific committee or a formal agreement between institutions. The request for approval guarantees the compliance of the projects with the General Data Protection Regulation (GDPR) on data protection and privacy in the EU and the European Economic Area (EEA) [21], although major differences exist between EU MS in the national interpretation and application of the GDPR.

Limitations of this study concern the identification and contact of survey respondents. Members of the JA InfAct were highly collaborative and assisted the research team in this task by forwarding the questionnaire to public health professionals engaged in health data management at national or international level. This is a convenience sampling method but it enabled the distribution of the survey instrument in all 28 MS and 4 associated countries. In addition, the selection of projects was subjective and by no means comprehensive. We realize that this approach may have resulted in a selection of the best performing data collections at the national and international level and that the observed compliance to quality and other standards may be a positively biased reflection of national practices. They also send the message, however, that it is well possible to engage in data sharing and international collaboration that result in more and better research output and policy support. Another limitation of the study regards data reusability; in particular, we did not address whether the different projects provided open access analytical pipelines for a full reuse of their methodologies, such as source codes and related documentation, analytical softwares, and more. We focused on data and metadata availability as the minimum requirement for research data reusability.

## Conclusions

The main challenges for health information in Europe, as identified in this study, are differences between and within countries in health data availability, accessibility, quality and comparability. Adherence to guidelines and protocols on standardized procedures in data collection, analysis and reporting is essential to ensure the comparability of research outputs. Likewise, adherence to EU policies on open data [20] and to FAIR Data Principles [11] are also fundamental in order to make data findable, accessible, interoperable, and reusable. A future European health information infrastructure could be an important step towards FAIR data use and could serve as a platform to foster exchange between researchers and research networks across and within the EU MS.

## Supplementary information

**Additional file 1.** Questionnaire for Member States regarding health data collection methods and procedures. Questionnaire used for the cross-sectional study

**Additional file 2.** Glossary of terms: Description of terms used in the survey questionnaire

**Additional file 3.** Projects identified through the survey on data collection methods and procedures. List of identified projects

## Data Availability

The datasets used and/or analysed during the current study are available from the corresponding author on reasonable request.

## Abbreviations

Atlas VPM: Spanish National Framework for Information Security for a geospatial project
BOD: Burden of Disease Network
CDC: Centers for Disease Control and Prevention
CHANCES: Consortium on Health and Aging-Network of Cohorts in Europe and the United States
CLSI: Clinical and Laboratory Standards Institute
EARS-Net: European Antimicrobial Resistance Surveillance Network
ECDC: European Centre for Disease Prevention and Control
ECHIM: European Community Health Indicator Monitoring System
ECHO: European Collaboration for Healthcare Optimization
EEA: European Economic Area
EHES: European Health Examination Survey
EHIS: European Health Interview Survey
EMCDDA: European Monitoring Centre for Drugs and Drug Addiction
EU: European Union
EUBIROD: European Best Information Through Regional Outcomes In Diabetes
EUROCAT: European network of population-based registries for the epidemiological surveillance of congenital anomalies
EUROCISS: European Cardiovascular Indicators Surveillance Set
EuroHOPE: European Health Care Outcomes, Performance and Efficiency
Euro-Peristat: European Perinatal Health Surveillance System
EURORDIS: Rare Diseases Europe
Eurostat: European Statistical Office
EUScreen: Vision and hearing screening programmes for children in Europe
FAIR: Findable, Accessible, Interoperable, and Reusable
GBD: Global Burden of Disease
GDPR: General Data Protection Regulation
HBSC: Health Behaviour in School-aged Children
HBM4EU: EU Network of Human Biomonitoring Laboratories
HM: Health Monitoring
HSPA: Health System Performance Assessment
IARC: International Agency for Research on Cancer
ICMJE: International Committee of Medical Journal Editors
IHE Europe: Integrating the Healthcare Enterprise
InfAct: Information for Action - Joint Action on Health Information
INSPIRE: Infrastructure for Spatial Information in Europe
JAF: Joint Assessment Framework on Health
MORGAM project: MOnica Risk, Genetics, Archiving and Monograph
MS: Member States
NESCAV: Nutrition, Environment and Cardiovascular Health
OECD: Organization for Economic Co-operation and Development
PASSI d’Argento: Surveillance system in the population over 64 years
PASSI: Progress by local health units towards a healthier Italy
REITOX: European information network on drugs and drug addiction
SHARE-ERIC: Survey of Health, Ageing and Retirement in Europe
WHO: World Health Organization
