# Correction to: Health data collection methods and procedures across EU member states: findings from the InfAct Joint Action on health information

**DOI:** 10.1186/s13690-022-00806-5

**Published:** 2022-02-14

**Authors:** Brigid Unim, Eugenio Mattei, Flavia Carle, Hanna Tolonen, Enrique Bernal-Delgado, Peter Achterberg, Metka Zaletel, Stefanie Seeling, Romana Haneef, Anne-Charlotte Lorcy, Herman Van Oyen, Luigi Palmieri

**Affiliations:** 1 Department of Cardiovascular, Endocrine-metabolic Diseases and Aging, Istituto Superiore di Sanità, Via Giano della Bella 34, 00162 Rome, Italy; 2 Center of Epidemiology, Biostatistics and Medical Information, Marche Polytechnic University, Ancona, Italy; 3 Department of Public Health and Welfare, Finnish Institute for Health and Welfare (THL), Helsinki, Finland; 4 Data Sciences for Health Services and Policy Research Group, Institute for Health Sciences in Aragon (IACS), Zaragoza, Spain; 5 Centre for Health Knowledge Integration, National Institute for Public Health and the Environment (RIVM), Bilthoven, Netherlands; 6 Health Data Centre, National Institute of Public Health, Ljubljana, Slovenia; 7 Department of Epidemiology and Health Monitoring, Robert Koch Institute, Berlin, Germany; 8 Department of Non-Communicable Diseases and Injuries, Santé Publique France, 94415 Saint-Maurice, France; 9 Directorate of Health, Luxembourg, Luxembourg; 10 Epidemiology and Public Health, Sciensano, Brussels, Belgium

**Correction to: Archives of Public Health (2022) 80:17**

**https://doi.org/10.1186/s13690-021-00780-4**

Following publication of the original article [1], the authors noticed that the legends of Figs. 2 and 3 were missing.

The complete Figs. 2 and 3 have been provided in this Correction.

**Fig. 2:**
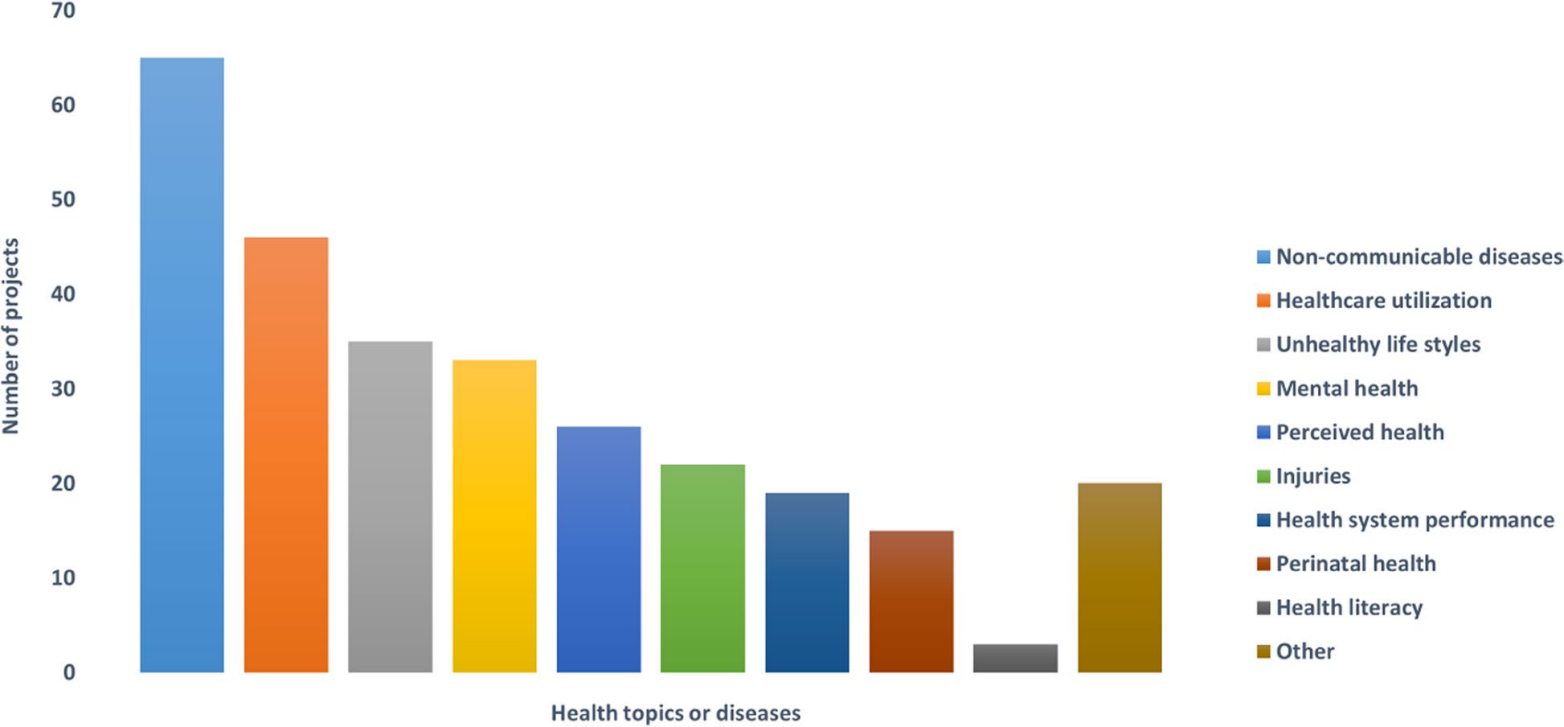
Health topics or diseases considered in the projects

**Fig. 3:**
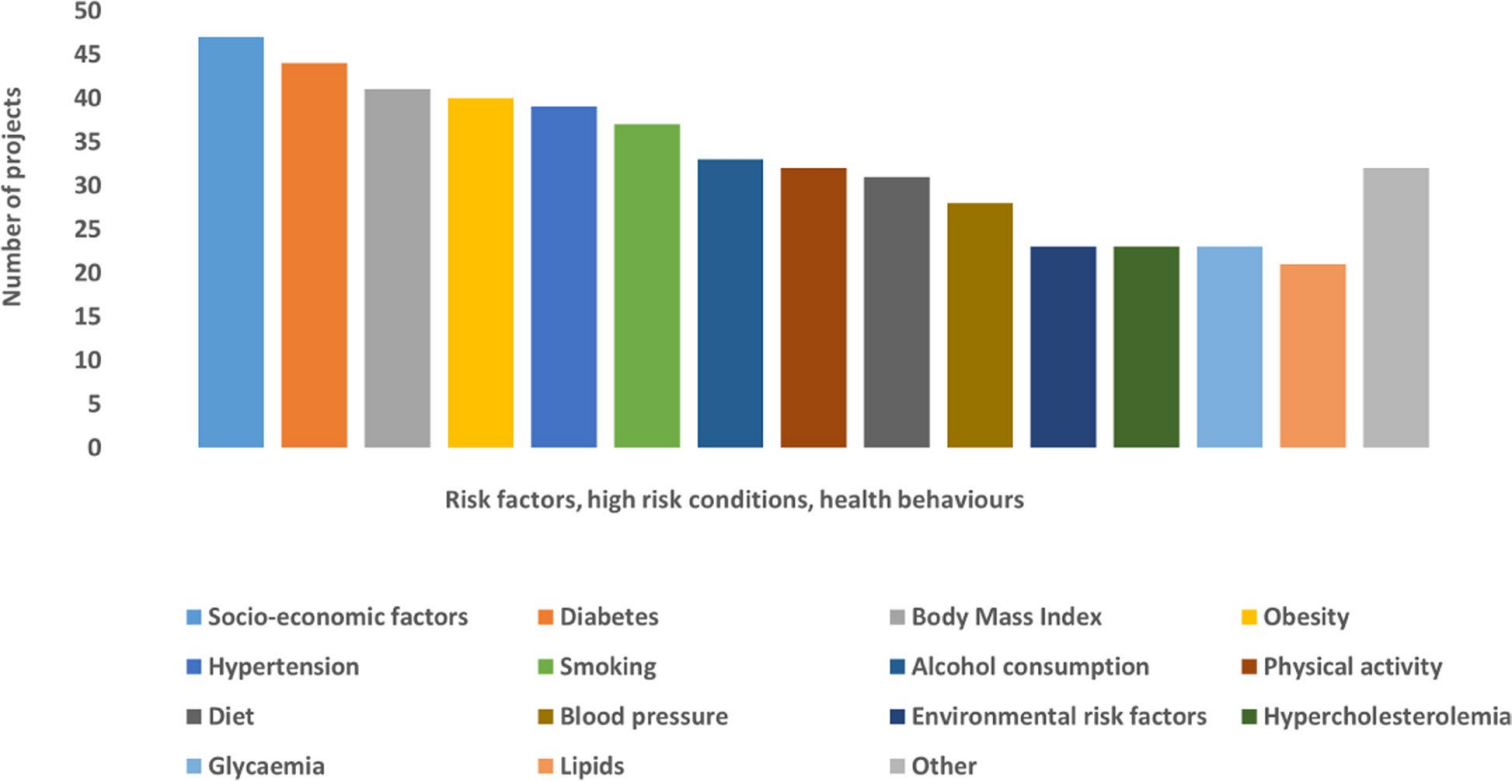
Risk factors, high-risk conditions and health behaviours investigated in the projects

The original article [1] has been updated.
