# Supplementary material for: Health data collection methods and procedures across EU member states: findings from the InfAct Joint Action on health information
Source: Arch Public Health. 2022 Jan 5;80:17. doi: 10.1186/s13690-021-00780-4 (PMC8728985; doi:10.1186/s13690-021-00780-4)
Supplement: Supplementary file 1 — Additional file 1. Questionnaire for Member States regarding health data collection methods and procedures. Questionnaire used for the cross-sectional study [file 13690_2021_780_MOESM1_ESM.pdf]

ADDITIONAL FILE 1. QUESTIONNAIRE FOR MEMBER STATES REGARDING HEALTH DATA COLLECTION METHODS AND PROCEDURES

1.GENERAL CHARACTERISTICS OF RESPONDENTS

1. Name of the country: ______________ (dropdown menu on the online version)

2. Last name of the contact person: ______________________ First name: ___________________

3. Type of institute:
□ Public Health Institute □ Research Institute
□ University □ National Statistics Department/Institute
□ International Organization □ Other, please specify _________________
□ Ministry of health/ Ministry of research

4. Work telephone number: ________________ 5. E-mail: ___________@_______________

2. SOURCE OF INFORMATION/DATA SOURCES - PROJECT/STUDY BACKGROUND INFORMATION

1. What is the name of the project/study: _____________________________
□ If available, provide a link to the website: _________________________________

2. Which authority/organization is responsible for this project/study:
____

3. Who is the contact person for this project/study:
Name: ______________________________________
E-mail address: _______________________________

4. The project/study is representative at:
□ regional level
□ national level
□ both

5. Which are the main objectives of the project/study (select all that applies)?
□ Health data collection
□ Elaboration of health monitoring indicators (e.g., prevalence, incidence, etc.)
□ Elaboration of health system performance assessment indicators (e.g., hospital-acquired infections, average length of stay, etc.)
□ Standardization and harmonization of methods and procedures
□ Development and/or validation of specific tools
□ Classifications and guiding principles
□ Other, please specify ___________________________

6. What type of health data sources are used (select all that applies)?
□ Population health examination survey (HES)
□ Population health interview survey (HIS)

□ Population-based disease registries
□ Hospital based registries
□ Clinical quality registries
□ Medical record or clinical data registries
□ e-health solutions (mhealth devices)
□ Longitudinal or cohort study
□ Administrative data (e.g., hospital discharge records, mortality, pharmaceutical prescription, etc.)
□ Electronic medical/health records
□ Intermediate linked data sources
□ Primary data collected by direct examination (DA AGGIUNGERE)
□ Primary data collected through interview (DA AGGIUNGERE)
□ Other, please specify ___________________________
¤ (available after each selected item in question 5). Please, specify the name of the health data source(s) and, if available, provide a link to the website

____

Please, specify if the data collection is:
□ Continuous
□ Periodic; please specify the interval ______________________________
□ Single implementation

7. What type of tools or approaches are used for the health data collection (select all that applies)?
□ Self-administered questionnaires
□ Face-to-face interviews
□ Telephone-based interviews
□ Direct examinations
□ Record linkage of various data sources
□ Electronic medical/health records
□ Mandatory reporting from data providers (i.e., administrative data collection)
□ Other, please specify _______________________

8. Are health data collected/used by the project/study shared with European research networks (e.g. EUROCISS, EHES, ECHIM, EUBIROD, ECHO, EuroREACH, etc.)?
□ Yes
□ No
□ Under development
¤ If yes, please specify the research network:
□ ________________________________
□ ________________________________
□ ________________________________

¤ If under development, please specify the research network:
□ _________________________________
□ _________________________________
□ _________________________________

9. How is the project/study funded (select all that applies)?
□ Public
□ Private
□ Other, please specify ________________________________

10. Please specify if the project/study is related to:
□ Health monitoring
□ Health system performance monitoring
□ Health system performance assessment

11. On which of the following main diseases or health topics did the project/study provide information (select all that applies)?
□ Non-communicable diseases (e.g., cardiovascular, cancer, pulmonary, diabetes, etc.)
□ Injuries
□ Unhealthy lifestyles
□ Mental diseases
□ Perinatal
□ Rare diseases
□ Perceived health
□ Health literacy
□ Health system performance
□ Healthcare utilization
□ Other, please specify ____________________________

12. On which of the following main risk factors, high-risk conditions and health behaviors did the project/study provide information (select all that applies)?
□ Blood pressure
□ Hypertension
□ Lipids
□ Hypercholesterolemia
□ Glycaemia
□ Diabetes
□ BMI
□ Obesity
□ Smoking
□ Alcohol consumption

□ Physical activity
□ Diet
□ Socio-economic factors
□ Environmental risk factors
□ Other, please specify ____________________________

13. Which of the following areas is defined in the project/study protocol (select all that applies):
□ Quality data control
□ Accessibility
□ Availability
□ Analysis
□ Reporting
□ Data linkage
□ Data sharing
□ Other, please specify _______________________________

¤ (after each selected item in question 13) Does the protocol include internationally recognized standardized methods and procedures for the selected areas?
□ Yes
□ No

¤ (available if "yes" for each selected item in question 13) Please, specify the reference or provide a link to the standardized methods and procedures ____

14. Which are the main indicators elaborated from the collected health data (select all that applies)?
□ Prevalence
□ Incidence
□ Attack rates
□ Performance measures
□ Outcome measures
□ Other, please specify __________________________

15. What is the main use of the elaborated indicators (select all that applies)?
□ Monitoring
□ Policy planning
□ Research
□ Health services evaluation
□ Other, please specify ____________________________

3. QUALITY ASSURANCE PROCEDURES IN DATA COLLECTION

The quality of statistical information is composed of the following dimensions or criteria: relevance, accuracy, timeliness and punctuality, comparability, coherence, accessibility and clarity (see glossary of terms).
1. Considering the above definition and the specific project/study indicated in section 2 - question 1, which dimensions or criteria are evaluated in quality assurance procedures at the national level (select all that applies)?
□ Relevance
□ Accuracy
□ Timeliness
□ Punctuality
□ Comparability
□ Coherence
□ Accessibility
□ Clarity
□ Coverage
□ Internal reliability
□ Other, please specify __________________________
□ All above mentioned dimensions or criteria
□ None of the above

2. For each of the selected data sources in section 2 - question 6, please provide your opinion/judgment regarding quality assessment of the health data in the table below.

Data source 1 (only those data sources selected in section 2 - question 6 will be shown)

□ Population health survey (HES)
□ Population health survey (HIS)
□ Population-based disease registries
□ Hospital based registries
□ Clinical quality registries
□ Medical record or clinical data registries
□ e-health solutions (mhealth devices)
□ Longitudinal or cohort study
□ Administrative data (e.g., hospital discharge records, mortality, pharmaceutical prescription, etc.)
□ Electronic medical/health records
□ Intermediate linked data sources
□ Other, please specify ________________________

Quality assessment criteria	Description*	Highly adequate	Adequate	Present but not adequate	Not adequate at all	Comments
Relevance	Degree to which statistics meet current and potential user needs					
Accuracy	Closeness of computations or estimates to the (unknown) exact or true values					
Timeliness	Length of time between its availability and the event or phenomenon it describes					
Punctuality	Time lag between the release date of data and the target date when it should have been delivered					
Comparability	Measure of the impact of differences between geographical areas, non-geographical domains, or over time					
Coherence	Adequacy to be reliably combined in different ways and for various uses					
Accessibility	Physical conditions under which users can obtain data					
Clarity	Availability of data information (documentation and metadata, illustrations, limitation in use, etc.)					
Coverage	The extent to which the sample stored describes actual performance.					
Internal reliability	A measure of whether the information stored is consistent over the years.					

*See glossary of terms.

▪ Data source n……. (If other data sources are indicated in section 2)

4. AVAILABILITY

1. Are the collected health data stored as micro (individual record) and/or macrodata (aggregated data)?
□ Microdata
□ Macrodata
□ Both

□ If microdata are available, is there a global unique and eternally persistent identifier (study identifier)?
□ Yes
□ No

□ If macrodata are available, is there an interactive system for users to perform further data aggregation and/or stratification?
□ Yes
□ No

2. Which are the available formats of the collected health data (select all that applies)?
□ Publication(s) (please specify the reference of relevant publication(s) ________________)
□ Electronic files
□ CD-ROM
□ Websites (please specify the link __________________)

3. Is there a publicly available description of the dataset purpose and content (metadata)?
□ Yes
□ No
□ If yes, please provide a web-link(s) to the public information
____

4. Do metadata follow reporting standards (e.g., SIMS, ESMS, ESMS-IP, ESQRS, OAIS, DDI described in Introduction section)?
□ Yes
□ No
□ I do not know/not aware

5. ACCESSIBILITY

1. Are the collected health data accessible to external users?
□ Yes, microdata
□ Yes, macrodata (aggregated data)
□ No

□ If "yes microdata", the data are
□ available to users upon specific request followed by approval
□ available to all users without specific request (open access)

□ If "yes macrodata (aggregated data)", the data are:
□ available to users upon specific request followed by approval
□ available to all users without specific request (open access)

If access is based on approval, how is the approval granted (select all that applies)?
□ By a scientific committee
□ By an ethics committee
□ Administrative committee
□ Legal committee
□ Formal agreement between institutions
□ Other (please specify): ________________________________

2. Are data reusable (i.e., data have a clear usage licenses and provide accurate information on provenance)?
□ Yes, for all users
□ Yes, based on data usage license (e.g., for a specific project, analysis, period of use, private or public use)
□ No
If "yes, for all users", please specify if macro or microdata.
If "yes, based on data usage license", please specify if macro or microdata.

3. Is there a remote data access service provided for users?
□ Yes
□ No
□ If yes, please provide the website address: ___________________________

4. Is there a financial charge for data access?
□ Yes
□ No

We thank you for your participation in this survey.
If you have another project/study to share with us, please click the following link: 'survey link'.
