# Supplementary material for: Health data collection methods and procedures across EU member states: findings from the InfAct Joint Action on health information
Source: Arch Public Health. 2022 Jan 5;80:17. doi: 10.1186/s13690-021-00780-4 (PMC8728985; doi:10.1186/s13690-021-00780-4)
Supplement: Supplementary file 2 — Additional file 2. Glossary of terms: Description of terms used in the survey questionnaire [file 13690_2021_780_MOESM2_ESM.pdf]

ADDITIONAL FILE 2. GLOSSARY OF TERMS

- **Data:** characteristics or information, usually numerical, that are collected through observations [1]
- **Dataset:** any organized collection of data. The data set lists values for each of the variables and for each member of the dataset [2]
- **Microdata:** consist of sets of records containing information on individual respondents or business entities. To protect the anonymity of respondents (persons, organizations), the access to microdata is restricted [3]
- **Macrodata:** data derived from microdata by statistics on groups or aggregates, such as counts, means, or frequencies [4]
- **Metadata:** explanatory texts documenting statistical data and providing summary information on definitions of populations, objects, variables, the methodology and quality and the statistical production process in general. A distinction is generally made between structural and reference metadata [2]:
    - ***Structural metadata*** are used to identify, formally describe or retrieve statistical data, such as dimension names, variable names, dictionaries, dataset technical descriptions, dataset locations, keywords for finding data etc. For example, structural metadata refer to the titles of the variables and dimensions of statistical datasets, as well as the units employed, code lists (e.g., for territorial coding), data formats, potential value ranges, time dimensions, value ranges of flags, classifications used, etc.
    - ***Reference metadata*** (sometimes called explanatory metadata) describe the contents and the quality of the statistical data from a semantic point of view. They include explanatory texts on the context of the statistical data, methodologies for data collection and data aggregation as well as quality and dissemination characteristics.
- **Metadata reporting standards:** the main reference metadata-reporting standards used by Eurostat [5]
    - SIMS (Single Integrated Metadata Structure)
    - ESMS (Euro SDMX Metadata Structure)
    - ESMS-IP (Euro SDMX Metadata Structure – Indicator Profile)
    - ESQRS (ESS Standard Quality Report Structure)

    There are also other metadata/data reporting standards facilitating the access and reuse of public information, such as:
    - Open archival information system (OAIS), specifies how to maintain, transfer and disseminate archival information across institutions, both metadata and data from public archives. The aim of this reference model is to acknowledge the actors, responsibilities/roles and procedures for the long-term maintenance of archival datasets considered public good [6]
    - Data Documentation Initiative (also known as DDI or DDI Metadata), an international standard only for metadata standardization in the case of micro data collected because of official statistics (surveys, questionnaires, etc.) conducted in National Statistics bodies [7].
- **Source of information/data sources:** specific datasets, metadata sets, databases or metadata repositories where data or metadata are available. According to the various ways in which data are collected, data sources can be distinguished in administrative, survey and registry sources [4]
- **Quality assurance procedures in data collection/data sources:** Eurostat [8] defines quality of statistical information in terms of the following dimensions or criteria: relevance, accuracy, timeliness and punctuality, comparability, coherence, accessibility and clarity.
    - ***Relevance*** is the degree to which statistics meet current and potential user needs. It refers to whether all statistics that are needed are produced and the extent to which concepts (definitions, classifications etc.) reflect user needs;

- ***Accuracy*** in the general statistical sense denotes the closeness of computations or estimates to the (unknown) exact or true values;

- ***Timeliness*** of information reflects the length of time between its availability and the event or phenomenon it describes;

- ***Punctuality*** refers to the time lag between the release date of data and the target date when it should have been delivered, for instance, with reference to dates announced in some official release calendar, laid down by regulations or previously agreed among partners;

- ***Comparability*** aims at measuring the impact of differences in applied statistical concepts and measurement tools/procedures when statistics are compared between geographical areas, non-geographical domains, or over time;

- ***Coherence*** of statistics is their adequacy to be reliably combined in different ways and for various uses. When originating from different sources, and in particular from statistical surveys of different nature and/or frequencies, statistics may not be completely coherent in the sense that they may be based on different approaches, classifications and methodological standards;

- ***Accessibility*** refers to the physical conditions under which users can obtain data: where to go, are access to data free or restrictive, etc.

- ***Clarity*** refers to the data's information environment whether data are accompanied with appropriate documentation and metadata, illustrations such as graphs and maps, whether information on their quality is also available (including limitation in use etc.) and the extent to which additional assistance is provided.

- **Other quality dimensions or criteria considered by ECHO are** [9]:
  - ***Coverage:*** measures the extent to which the sample stored describes actual performance. Also represents a measure of the potential relevance of the data stored.
  - ***Internal reliability:*** a measure of whether the information stored is consistent over the years. It is a necessary condition for accurate estimations.
- **Availability:** availability of micro or macro data, in various formats (publications, files, CD-ROM, Internet, etc.) and documentation related to various aspects of the data, such as methodological documents, summary notes or papers covering concepts, scope, classifications and statistical techniques [8, 10]
- **Remote data access service:** a service providing access to data stored on a computer or network from a remote distance. Remote data access services are often secured to ensure that users can only access data to which they have been approved and that users cannot alter or withdraw/copy the data from the system without permission [11]
- **Health Examination Survey (HES):** population based and objective surveys that provide data on many health indicators to support policy making, preventive activities and research. HES include questionnaire about socio-economic, demographic and health issues, as well as objective physical measurements, such as weight and blood pressure, and collection of biological samples, such as blood or urine [12]
- **Health Interview Survey (HIS):** collection of health status, healthcare use, health determinants and socio-economic background variables of a representative sample of the population living in private households through standardized questionnaires. The European Health Interview Survey (EHIS) includes information from all European Union Member States and is to be conducted every five years. EHIS is used as a data source for important health and social policy indicators such as the European Core Health Indicators (ECHI) [13]
- **Population-based disease registry/register:** in epidemiology, the term register is applied to the file of data concerning all cases of a particular disease or other health-relevant condition in a

defined population such that the cases can be related to a population base. With this information, incidence rates can be calculated. If the cases are regularly followed up, information on remission, exacerbation, prevalence, and survival can also be obtained. The register is the actual document, and the registry is the system of ongoing registration [14]

- **Hospital-based disease registries** contain data on all patients with a specific type of disease diagnosed and treated at that hospital (e.g., cancer registries). There are two sub-categories under hospital-based registries: single hospital registries and multi-institution registries. The primary goal of the single hospital (institution) registry is to improve patient care by medical audit-type evaluation of outcomes [15]
- **Drug registries** (e.g., AIFA) record drugs and therapeutic plans submitted to monitoring [16]
- **Medical records** or **clinical data registries (e.g., Health search project, Pedianet project)** contain data on diagnoses, prescriptions and health assessments performed during each encounter with the patient and are recorded as part of the daily practice of physicians [17]
- **Clinical quality registries** (e.g., Sweet project, Pediatric Diabetes 2016) are organizations which systematically monitor the quality (appropriateness and effectiveness) of healthcare, within specific clinical domains, by routinely collecting, analyzing and reporting health-related information. They then feed this information back to clinicians to inform clinical practice and decision making [18]
- **Administrative source:** register of units and data associated with an administrative regulation (or group of regulations), viewed as a source of statistical data [2]
- **Survey:** investigation about the characteristics of a given population by means of collecting data from a sample of that population and estimating their characteristics through the systematic use of statistical methodology [2]
- **Longitudinal or cohort study:** observation of the population for a sufficient number of person-years to generate reliable incidence or mortality rates in the population subsets. This generally implies study of a large population, study for a prolonged period (years), or both [14]
- **e-health solutions:** e-health is an emerging field in the intersection of medical informatics, public health and business, referring to health services and information delivered or enhanced through the Internet and related technologies. In a broader sense, the term characterizes not only a technical development, but also a commitment to improve healthcare locally, regionally, and worldwide by using information and communication technologies [19]. Examples of e-health solutions are: i) electronic medical records or electronic health records; mobile health devices (mHealth) collecting survey data, ii) mobile payment processing technology to purchase fruits and vegetables; iii) EATFRESH.ORG is a healthy eating resource that offers multilingual information via its website, social media, and mobile technology; iv) Find MI Care is a free website and mobile application that simplifies the task of finding local, low-cost healthcare [20]
- **Healthcare performance measures**: measures that are commonly used to assess population health in relation to health-care performance. The measures focus on health insurance data as measure of occurrence, disease costs, or on patient data for quality assessment [21]
- **Indicator:** quantitative or qualitative factor or variable that provides a simple and reliable means to measure achievement, to reflect the changes connected to an intervention, or to help assess the performance of a development actor [22]
- **Intermediate linked data source:** a database in which individual information from different sources are linked to contextual information (namely, demographic statistic, socioeconomic data and information on supply) to produce intermediate outputs or data that can be further elaborated [9]

REFERENCES

1. OECD glossary on statistical terms. https://stats.oecd.org/glossary/detail.asp?ID=532
2. Eurostat glossary. http://ec.europa.eu/eurostat/statisticsexplained/index.php/Category:Statistical_concept
3. OECD Glossary of statistical terms. https://stats.oecd.org/glossary/detail.asp?ID=1574
4. OECD: glossary of statistics terms. htpps://stats.oecd.org
5. EUROSTAT. ESS reference metadata reporting standards. https://ec.europa.eu/eurostat/data/metadata/metadata-structure
6. Open Archival Information System (OAIS). https://www.iso.org/standard/57284.html
7. Data Documentation Initiative. https://www.ddialliance.org/explore-documentation
8. Eurostat: Standard Quality Report. Methodological Documents, Working Group "Assessment of quality in statistics", Luxembourg, 2-3 October 2003
9. European Collaboration for Healthcare Optimization (ECHO) Project. Zaragoza (Spain): Instituto Aragonés de Ciencias de la Salud - Instituto Investigación Sanitaria Aragón; c2010. Estupiñán Romero FR, Baixauli Pérez C, Bernal-Delgado E on behalf of the ECHO consortium. Handbook on methodology: ECHO information system quality report; 2014
10. SDMX Guidelines. SDMX glossary version 2.0, October 2018. https://sdmx.org/
11. OECD Health Statistics 2018: Frequently Requested Data (Excel file). Glossary of terms. http://www.oecd.org/health/publicationsdocuments/datasets/
12. European Health Examination Survey: National health examination surveys in Europe. http://www.ehes.info/national_hes.htm
13. Eurostat glossary: European health interview survey (EHIS). https://ec.europa.eu/eurostat/statistics-explained/index.php?title=Glossary:European_health_interview_survey_(EHIS)
14. Porta M, Greenland S, Last JM (eds): A Dictionary of Epidemiology. Oxford University Press, Oxford, Fifth edition, 2008
15. National Cancer Institute. SEER training Modules: Hospital-Based Registries. https://training.seer.cancer.gov/registration/types/hospital.html
16. Montilla S, Xoxi E, Russo P, Cicchetti A, Pani L. Monitoring registries at italian medicines agency: fostering access, guaranteeing sustainability. Int J Technol Assess Health Care 2015;31(4):210-3
17. National Quality Registry Network: What is a Clinical Data Registry? https://www.abms.org/media/1358/what-is-a-clinical-data-registry.pdf
18. Australian commission on safety and quality in healthcare. https://www.safetyandquality.gov.au/our-work/information-strategy/clinical-quality-registries/
19. Eysenbach G. What is e-health? J Med Internet Res. 2001; 3(2): e20
20. The Promises and Perils of Digital Strategies in Achieving Health Equity: Workshop Summary. Roundtable on the Promotion of Health Equity and the Elimination of Health Disparities; Board on

Population Health and Public Health Practice; Health and Medicine Division; National Academies of Sciences, Engineering, and Medicine. Washington (DC): National Academies Press (US); 2016

21. Smith PC, Mossialos E, Papanicolas I, Leatherman S. Performance Measurement for Health System Improvement: Experiences, Challenges and Prospects. WHO Europe/European Observatory on Health Systems and Policies, 2009
22. Glossary of the main terms used in results based management. www.oecd.org/development/peer-reviews/17484948.pdf
