# Supplementary material for: Health data collection methods and procedures across EU member states: findings from the InfAct Joint Action on health information
Source: Arch Public Health. 2022 Jan 5;80:17. doi: 10.1186/s13690-021-00780-4 (PMC8728985; doi:10.1186/s13690-021-00780-4)
Supplement: Supplementary file 3 — Additional file 3. Projects identified through the survey on data collection methods and procedures. List of identified projects [file 13690_2021_780_MOESM3_ESM.pdf]

ADDITIONAL FILE 3. PROJECTS IDENTIFIED THROUGH THE SURVEY ON DATA COLLECTION METHODS AND PROCEDURES

COUNTRY	PROJECT	RN
**Belgium**	Belgian Treatment Demand Indicator Register	
**Belgium**	European Health Examination Survey	x
**Belgium**	Evaluation of ambulatory care quality	
**Belgium**	Health Interview Survey	x
**Belgium**	Health Status Report	
**Belgium**	Initiative for Quality Improvement and Epidemiology in Children and Adolescents with Diabetes	
**Belgium**	Initiative for Quality improvement and Epidemiology in Diabetes	
**Belgium**	Initiative for Quality improvement and Epidemiology in multidisciplinary Diabetic Foot Clinics	
**Croatia**	CroDiab	
**Czech Republic**	European Health Examination Survey	x
**Estonia**	The health insurance coverage study	
**Finland**	Different administrative registries	
**Finland**	Finland Health survey	
**Finland**	FinSote	
**France**	Euro-Peristat	x
**France**	Surveillance of cardiovascular diseases	
**Germany**	AdiMon Indicator System	
**Germany**	BURDEN 2020	x
**Germany**	Health Interview and Examination Survey for Adults	x
**Germany**	Health Interview and Examination Survey for Children and Adolescents	
**Germany**	Health Update - GEDA	
**Germany**	National diabetes surveillance	
**Italy**	A plan for Evaluating Costs and Outcomes of colorectal Surgery in Emilia-Romagna (Emilia-Romagna Surgical Colorectal cancer Audit-ESCA)	
**Italy**	CAMUNI cerebrovascular disease registry	
**Italy**	CAMUNI Registry of Myocardial Infarctions	
**Italy**	CARENET - Performance evaluation and value assessment for cardiovascular and oncological care path in a regional network context: challenges and opportunities	x
**Italy**	COACH - Comparing Outcomes of Acute Cerebrovascular and other neurological Hospitalizations	
**Italy**	Developing and validating a new population-based risk stratification tool for predicting mortality, hospital admissions and healthcare costs	
**Italy**	Developing and validating a novel multisource comorbidity score from administrative data: a large population-based cohort study from Italy.	
**Italy**	Drug-related mortality and hospitalization in Italy	
**Italy**	Epidemiological Surveillance	
**Italy**	EU-ADR - Exploring and understanding adverse Drug reactions by integrative mining of clinical records and biomedical knowledge	
**Italy**	European Injury Database	x

**Italy**	FABIO - Valutazione dell'utilizzo di FArmaci BIOlogici nel pazienti Oncologico	
**Italy**	FRAME - Flussi Regionali Automatizzati per il Monitoraggio dell'assistenza e la generazione di Evidenze scientifiche di indirizzo per le politiche sanitarie	x
**Italy**	GIRO - Gruppo Italiano Reti Oncologiche	x
**Italy**	GRETA - Generating Real-world Evidence on the Treatment of metastatic colorectal cancer with Avastin-bevacizumab	
**Italy**	Health Behaviour in School-aged Children (HBSC)	x
**Italy**	Improving microbiology diagnostic system quality in the function of surveillance on communicable diseases in the Republic of Serbia	
**Italy**	IST-02566 Differenze di mortalità e di ospedalizzazione secondo lo stato di salute, gli stili di vita e il consumo di servizi sanitari	
**Italy**	Italian Longitudinal Study on Aging - ILSA	x
**Italy**	Italian nationwide longitudinal population-based study on DKA at diagnosis of type 1 diabetes	x
**Italy**	Italian Obstetric Surveillance System (ItOSS)	x
**Italy**	Italian PRoject on the Epidemiology of Alzheimer's disease - IPREA	x
**Italy**	LINFA Project: Longitudinal Infant and Neonatal Follow-up towards Adolescence	
**Italy**	MACHINE - Mother And CHild-INfant real-world Experience	
**Italy**	Moli-sani Study	
**Italy**	MONICA-Brianza	x
**Italy**	National Registry of Major Coronary and Cerebrovascular Events	
**Italy**	Surveillance system OKkio alla SALUTE	
**Italy**	Surveillance system Passi d'argento	
**Italy**	Patterns of multimorbidity	
**Italy**	Pharmacological treatment in the elderly patient affected by cardiovascular disease and other chronic comorbidities: inappropriate prescribing and outcome evaluation among institutionalized and community-dwelling elders	
**Italy**	Population-based specialized gastric cancer registry in the province of Cremona	
**Italy**	Profili di salute	
**Italy**	Progetto PDTA - Metodologia per il monitoraggio e la valutazione dei percorsi diagnostico-terapeutico assistenziali (PDTA) nell'ambito del Nuovo Sistema di Garanzia dell'assistenza sanitaria	
**Italy**	QUADIM - I percorsi di cura nei disturbi mentali gravi, tra valutazione della qualità della cura e nuovi modelli di finanziamento	
**Italy**	Risk of Cardiovascular diseases and abdominal aortic Aneurysm in Varese (RoCAV)	
**Italy**	SAFEGUARD – Safety Evaluation of Adverse Reactions in Diabetes	
**Italy**	Socio-economic inequalities in mortality	
**Italy**	SOS - Safety of non-steroidal anti-inflammatory drugs	
**Italy**	Surveillance system PASSI	
**Italy**	The Viadana study	
**Italy**	Valutazione degli eventi avversi (cardio e cerebrovascolari) negli utilizzatori di incretine e altri antidiabetici attraverso l'analisi dei database amministrativi della Regione Lombardia	
**Italy**	Web-based antimicrobial surveillance tool	
**Latvia**	Health Care Monitoring Datalink	
**Luxembourg**	Carte sanitaire	
**Luxembourg**	European Health Examination Survey	x
**Luxembourg**	European Injury Database	x
**Luxembourg**	Health Behaviour in School-aged Children (HBSC)	x

**Luxembourg**	Luxembourg's Birth-Related Health-Monitoring System - SUSANA	
**Luxembourg**	Luxembourgish Information System on Drugs and Drug Addiction	
**Luxembourg**	Neonatal Hearing Screening	
**Luxembourg**	Observation of Cardiovascular risk factors in Luxembourg - ORISCAV-LUX 1 &2	
**Luxembourg**	SHARE - Survey of Health, Ageing and Retirement in Europe	x
**Luxembourg**	Study of infant feeding practices for babies aged 4, 6 and 12 months in Luxembourg	
**Netherlands**	Doetinchem Cohort Study	
**Netherlands**	Nivel Primary Care Database	
**Portugal**	National Health Interview Survey	x
**Romania**	Romanian study	
**Serbia**	Monitoring of health care quality indicators	
**Slovenia**	CINDI Health Monitor Survey	x
**Slovenia**	National Dietary Survey (EU-MENU)	
**Slovenia**	National Survey on Oral Health	
**Slovenia**	Registry of sick-leave (from work)	
**Slovenia**	Registry on Causes of Deaths	
**Slovenia**	Study on incidence and prevalence of diabetes	
**Slovenia**	Survey on use of alcohol, tobacco and illicit drugs	
**Spain**	Atlas of Variations in Medical Practice in the Spanish National Health Service (Atlas VPM project)	
**Sweden**	The National Public Health Survey	
**United Kingdom**	Secure Anonymized Information Linkage (SAIL) system	

RN, Research Network
